# Impact of Droplet Splashing on Biological and Medical Materials Relevant to Clinical Settings

**DOI:** 10.1002/smsc.202400239

**Published:** 2025-05-30

**Authors:** Mohammad Hadi Esteki, Ian Eames, Emad Moeendarbary

**Affiliations:** ^1^ Department of Mechanical Engineering University College London Torrington Place London WC1E 7JE UK; ^2^ Department of Mechanical Engineering Isfahan University of Technology Isfahan 8415683111 Iran

**Keywords:** advanced biomaterials, biological substrates, droplet impact, infection control, secondary droplets, splashing dynamics, surface roughness

## Abstract

Despite numerous studies of droplet impact onto substrates, the splashing dynamics of droplets on biological material surfaces and its implications for infection transmission have rarely been studied. It is hypothesized that the splashing mechanism is influenced by the droplet size, the impact velocity, and the substrate wettability and morphology. The transmission of contamination from initial droplets or liquid films to biofilms upon impact is experimentally investigated. Splashing mechanisms involving biological droplets (e.g., water, urine, blood, and saliva) on a range of biological substrates (e.g., bone, meat, eye, skin, hair, nail, and tooth) and medical surfaces across a range of droplet velocities are comparatively analyzed. The study demonstrates that contaminants located in either an initial droplet or a liquid biofilm can be transmitted by splashing when the droplet impacts onto the biofilm. The Weber number, a descriptor of secondary droplet splashing, expressed as a function of the surface roughness (*R*_a_) is considered. For a droplet of radius *R*, the prominence of the surface curvature (*R*_a_ */R*) is highlighted through comprehensive experimentation, underscoring the importance of using tailored surface materials within clinical environments.Developing advanced biomaterials and designs can thus help reduce droplet splashing and promote safer medical procedures.

## Graphical abstract

Droplet splashing on biological and medical materials poses contamination risks in clinical settings. Using high‐speed imaging and quantitative modelling, this study reveals how fluid properties and surface features—like roughness and curvature—govern splash dynamics. The findings inform design strategies for advanced biomaterials to reduce secondary droplet formation and enhance infection control.

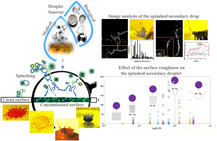

## 1 Introduction

**Figure 1:**
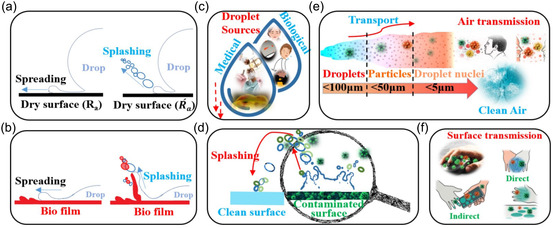
Schematic illustration of propagation mechanisms and infectious droplet transmission in medical environments. a,b) Spreading and splashing dynamics upon droplet impact onto a dry surface or a biofluid layer, respectively. c) Illustrated provenance of droplets: biological and medical origins. d) Illustrated representation of the study focus: splashing of a contaminated secondary droplet and its air‐ or surface‐based dispersal. e) Visualizing aerosol and droplet transport via splashing and its implications for airborne transmission. f) Mapping droplet transport through splashing: direct and indirect surface transmission.

The splashing of droplets onto substrates has generated substantial interest in recent years because of its clinical and research implications.^[^ ^1^, ^2^, ^3^, ^4^, ^5^, ^6^, ^7^ ^]^ This phenomenon is particularly relevant in the contexts of bacterial and viral disease propagation through air or of surface contamination.^[^ ^8^, ^9^ ^]^ The development of methods for reducing droplet‐based disease transmission requires an understanding of droplet splashing dynamics, especially in medical and biological settings that frequently employ high‐power mechanical devices and involve high outflow pressures.^[^ ^9^, ^10^, ^11^ ^]^ Procedures used in orthopedics, otorhinolaryngology, oculofacial plastic surgery, laparoscopy, ophthalmology, cesarean delivery, and dentistry all utilize such powered devices or high outflow pressures, potentially causing biofluid leakage to surrounding areas.^[^ ^9^, ^10^, ^11^, ^12^, ^13^, ^14^, ^15^, ^16^, ^17^, ^18^, ^19^ ^]^ Biofluids such as blood, saliva, mucus, urine, vomit, and tears can also transmit bacterial and viral infections.^[^ ^20^, ^21^, ^22^, ^23^, ^24^, ^25^, ^26^, ^27^ ^]^ **Figure** **1** schematically portrays key aspects of the splashing mechanisms and contamination displayed by droplets. Figure 1a,b illustrates the basic behaviors (spreading and splashing) displayed when a droplet impacts onto either a solid surface or a biofilm layer, respectively. Figure 1c illustrates the diverse sources of the droplets we examined in the present study, differentiating between biological droplets originating from, e.g., coughing and sneezing and those generated by medical devices and procedures. Figure 1d zooms in on the central subject of this study, namely, the splashing of secondary droplets that, when contaminated, contribute to surface and airborne transmissions.

Figure 1e,f depicts droplet transport through splashing, highlighting its role in both airborne and surface transmission. Various studies have highlighted the significance of droplet dynamics and aerosol behavior in contexts that include respiratory virus transmission, healthcare settings, surgical procedures, and endoscopy infection control. They emphasize the importance of understanding droplet behavior to mitigate the disease transmission risk, particularly in situations involving high outflow pressures and high‐power mechanical devices.^[^ ^3^, ^5^, ^6^, ^7^, ^8^, ^9^, ^10^, ^11^, ^12^, ^20^, ^27^, ^28^, ^29^ ^]^ Previous studies investigated various aspects of bioaerosols, including contaminated droplets, in relation to disease transmission, highlighting their significance in healthcare settings and emphasizing the need for further research.^[^ ^28^, ^30^, ^31^, ^32^ ^]^ Although the current study did not explicitly quantify the aerosol fraction of the splashed droplets, the experimental conditions and observed droplet fragmentation suggest that aerosol‐sized droplets could form.

Droplet behavior upon impact is frequently described using the Weber number, defined as We = *ρDV* ^2^ */σ*, where *ρ* is the fluid density, *D* is the droplet diameter, *V* is the impact velocity, and *σ* is the surface tension. Worthington pioneered the study of splashing phenomena resulting from liquid droplets impacting onto a solid or liquid surface, leading to a wide range of applications for material processing, ink printing, and irrigation.^[^ ^33^, ^34^, ^35^, ^36^, ^37^ ^]^ Nevertheless, the underlying splashing mechanism remains only partially understood. Various factors, including the droplet properties, their surface characteristics, and environmental variables, intricately govern the splashing process. Key determinants, such as the droplet size, the impact velocity, and the substrate wettability and geometry, significantly influence the splashing behavior.^[^ ^38^, ^39^, ^40^, ^41^, ^42^ ^]^ The calculation of secondary droplet velocities employs diverse methods, including empirical correlations (e.g., using the Rosin–Rammler equation), numerical simulations through computational fluid dynamics, experimental measurements using high‐speed videos and image analysis, and dimensional analysis.^[^ ^43^, ^44^, ^45^, ^46^, ^47^ ^]^ These multidisciplinary approaches collectively enhance our understanding of droplet splashing physics, offering valuable insights for practical applications such as spray coating, combustion control, and environmental assessments.^[^ ^48^, ^49^, ^50^, ^51^, ^52^ ^]^

The detailed interplay between substrate characteristics and splashing behavior provides valuable insights for designing surfaces that minimize splashing, particularly in the context of critical healthcare applications. The search for bioinspired strategies for preventing adverse phenomena has highlighted the importance of surface characteristics, including flexibility.^[^ ^53^ ^]^ Studies investigating contact‐angle hysteresis reveal how substrate properties, such as flexibility and roughness, affect wetting and droplet behavior.^[^ ^54^ ^]^ Investigations into antiwetting, self‐cleaning, and antibacterial surfaces in medical devices have established the essential role of substrate properties in healthcare applications, including splashing control.^[^ ^55^ ^]^ Research into wetting behavior on chemically patterned surfaces explores how the surface chemistry and substrate properties influence wetting transitions, an issue relevant to splashing control in various scenarios.^[^ ^56^ ^]^ Reviews on surface engineering for orthopedic implants emphasize the importance of substrate characteristics in medical‐device design and for preventing infections, highlighting strategies that minimize splashing and contamination risks in healthcare settings.^[^ ^57^ ^]^

The dynamics of a droplet impacting onto a solid surface is a multifaceted phenomenon. The outcomes of the component interactions are crucially determined by several factors, which include the liquid properties (e.g., the viscosity and surface tension), the impingement velocity, and the surface conditions (e.g., the wettability and roughness).^[^ ^2^, ^39^, ^58^, ^59^, ^60^, ^61^, ^62^, ^63^, ^64^ ^]^ The likelihood of splashing is determined by a delicate balance between, on the one hand, hydrodynamic and hydrostatic pressures that drive the radial spreading of the droplet upon impact, and on the other hand, opposing forces like the capillary pressure at the rim edge and the viscous stress. Splashing occurs when the driving forces exceed the opposing forces. This condition is strongly influenced by surface characteristics such as the wettability and surface roughness.^[^ ^65^, ^66^, ^67^ ^]^ In the context of droplet impact dynamics, the shape of the solid substrate fundamentally determines the splashing thresholds. Previous studies have utilized high‐speed imaging to observe millimetric droplets impacting onto surfaces across a wide range of substrate geometries and impact conditions.^[^ ^59^, ^67^, ^68^, ^69^ ^]^ Models of splashing have been used to investigate the splashing velocity (*v*_sp_) as a function of the fluid properties and the ejection time (*t*_e_). The revised quadratic equation indicates that *v*_sp_ is affected by the viscosity, density, surface tension, and droplet radius. On highly wettable surfaces, an increase in the ejection time leads to a decrease in *v*_sp_, resulting in a greater energy loss and reduced splashing. In contrast, on hydrophobic surfaces, shorter ejection times yield a higher *v*_sp_, raising the chances of splashing. Blood displays a slight deviation from this behavior owing to its non‐Newtonian characteristics while preserving the overarching trend associating a higher wettability with lower *v*_sp_.^[^ ^67^ ^]^ Recent studies have explored droplet impact dynamics extensively, emphasizing the influence of the surface properties and fluid characteristics on the spreading and splashing behavior. Riboux and Gordillo^[^ ^70^ ^]^ developed a predictive model for the critical impact velocity of a droplet splashing onto smooth surfaces, demonstrating the interplay between the impact velocity and ejection time. Their findings, further extended by De Goede et al.^[^ ^67^ ^]^ incorporated the effects of fluid viscosity and non‐Newtonian behavior, particularly in biological fluids such as blood. Additional investigations by De Goede et al.^[^ ^67^ ^]^ examined the role of surface roughness, revealing that rough substrates significantly alter splashing thresholds and dynamics. Building on these foundational works, our study not only validates these models but also introduces substrate‐specific predictive functions for the spreading area and velocity, accounting for hydrophilic and hydrophobic surface characteristics. Research into droplet impact dynamics has expanded to include biological fluids like blood, with studies highlighting the effects of substrate characteristics, the droplet size, and surface roughness to analyze bloodstain patterns, showcasing the practical implications of droplet impact studies.^[^ ^71^, ^72^, ^73^, ^74^ ^]^ Building on prior works, our study focuses on water droplet interactions, offering quantitative models for spreading and splashing dynamics, with potential extensions to non‐Newtonian fluids. These findings provide a comprehensive framework for understanding droplet behavior across diverse surface and fluid conditions, bridging gaps in current knowledge and opening new practical application possibilities in fields such as biomedicine and materials science.

Despite the detailed studies performed to date, our physical understanding of the role played by biological materials in generating splash droplets remains limited, even under typical in vitro conditions. The choice of solutions available for mitigating secondary droplet splashing, particularly through the utilization of biomaterials, is also limited. The aim of the present study was to help fill this knowledge gap by providing a more comprehensive characterization of the splashing behavior of various biological fluids. By utilizing high‐speed cameras and image processing, we experimentally investigated the splashing of fluids, such as horse blood, human urine, and saliva, and the spreading of water droplets upon impact with different material substrates, particularly those relevant to medical environments. Our objectives included the following: understanding the influence of contamination on splashing; conducting a comparative analysis of the splashing mechanisms involved for different biological droplets and substrates for varying droplet velocities and substrate flexibilities; and exploring the We of secondary splashing droplets with regard to the surface roughness, the substrate curvature (convex or concave), and its effect on the droplet velocity. Spreading models were utilized to predict the spreading velocity and area by using curve fitting. Both the spreading and splashing models yield good predictions of the spreading velocity and the spreading area for each surface, demonstrating the robustness of the spreading model. This can hence provide insights relevant to healthcare applications, particularly in the context of infection control using advanced biomaterials.

## 2 Experimental Section

### 2.1 Experimental Setup

**Figure 2:**
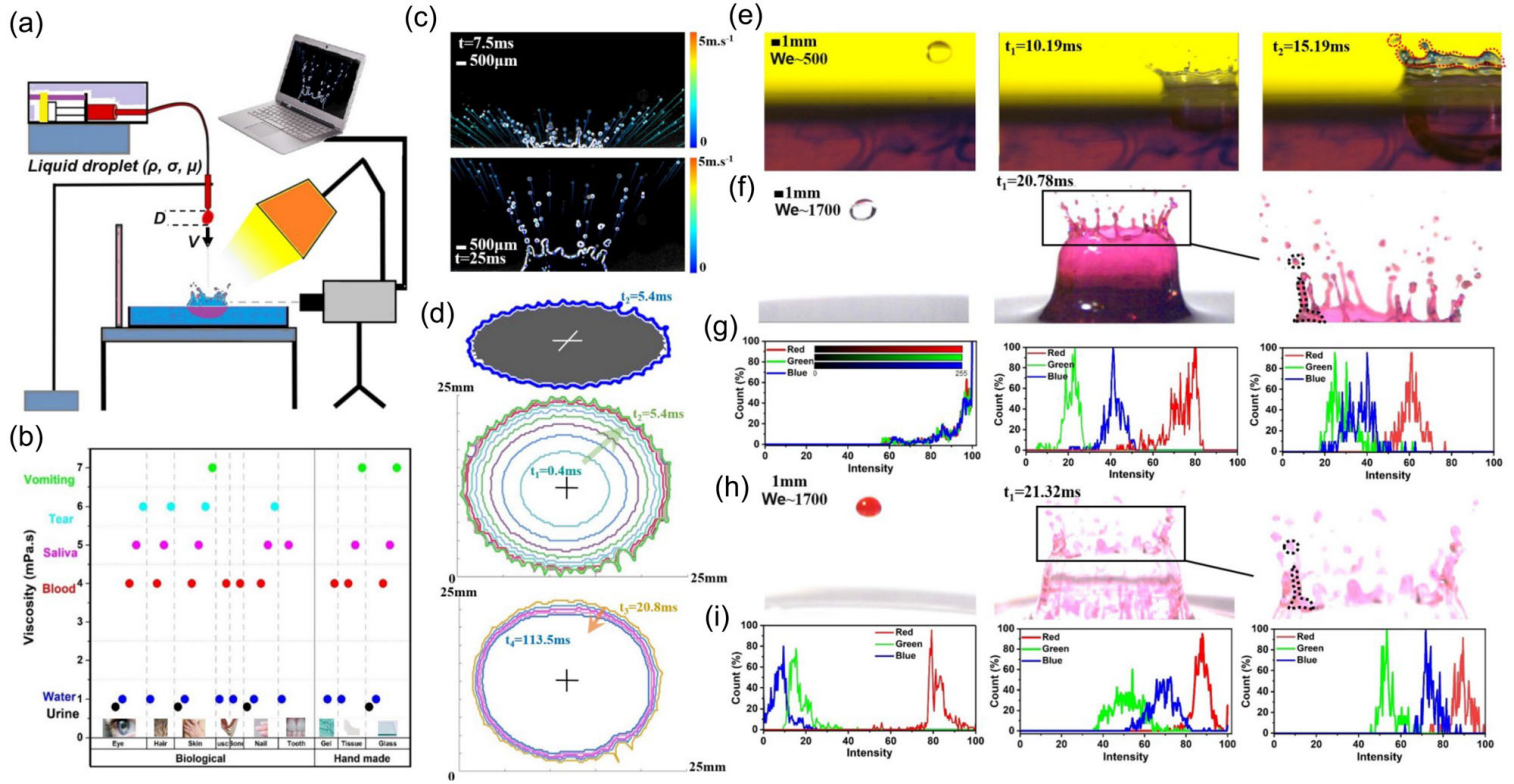
Experimental investigation and analysis of droplet impact and splashing onto a biofilm or biological substrate. a) Experimental setup: syringe pump, high‐speed camera, computer, and lighting. b) Comparison of the viscosity of different biofluids on various biological and nonbiological substrates. c) Processed images of a water droplet impacting onto a water surface. The velocity vectors of the secondary droplets produced by the splashing are given at times of 7.5 and 25 ms after impact. d) Processed images of a water droplet impacting onto a dry substrate, with the subsequent spreading until relaxation occurs. High‐resolution images of splashing events: e) colored secondary droplets generated from a colored water film; f) a noncolored droplet impacting onto a colored water layer; h) a colored droplet impacting onto a noncolored water layer, resulting in secondary colored droplets. Probability distributions of the color channels in (f) and (h) are plotted in (g) and (i), respectively, as functions of the corresponding pixel intensities.

The overall experimental concept is summarized in **Figure** **2**. The experimental setup, outlined in Figure 2a, includes the substrate platform, a high‐speed imaging system, a computer, a lighting system, and a droplet generator. Droplets are produced by a needle linked to a syringe pump. Experimental runs were conducted at 25 °C. Each impact event was repeated at least 5 times. The high‐speed imaging system consists of a high‐speed camera (PHANTOM VEO 710) and light sources suitable for imaging at 24 000 frames per second with a spatial resolution of 8.5 pixel mm^−1^ (pixel resolution 512 × 512).

### 2.2 Substrate Preparation

The biological and nonbiological substrates required meticulous preparation prior to the imaging measurements. The substrate preparation was tailored to the specific properties and characteristics of each substrate category: biofilm fluids and biological and nonbiological substrates.

To ensure reproducibility and provide a comprehensive understanding of the substrate preparation process, the selection, preparation, and characterization of the materials were conducted under carefully controlled laboratory conditions. The laboratory environment was maintained at 25 °C with a relative humidity of 50% and an atmospheric pressure of 1013 kPa to simulate standard ambient conditions. All experiments were carried out in a clean‐room environment (ISO 7) to minimize particulate contamination.

The biofilm fluid preparation involved sourcing the respective biological samples and processing them to create homogeneous fluids. Human urine and saliva were collected in a controlled environment and filtered using a sterile 0.45 μm membrane to remove particulate matter and contaminants. For horse blood, stringent sterile procedures were followed to obtain a consistent and uncontaminated fluid sample.^[^ ^75^ ^]^ These fluids were then used as impact targets to study the interactions of droplets with biological substrates. Figure 2b compares the viscosities of various biofluids including water, human urine, saliva, vomit, and horse blood on the different biological substrates, highlighting the influence of the rheological properties on droplet behavior within our experimental setup.^[^ ^76^, ^77^, ^78^, ^79^, ^80^, ^81^ ^]^

Nonbiological substrates were prepared by surface conditioning and uniformization to facilitate the performance of controlled experiments. Glass, plastic, wooden, and metal panels were meticulously cleaned and polished to eliminate surface irregularities or contaminants. Tissue paper samples of varying textures were chosen. A standard nurse uniform was subjected to standardized cleaning procedures. Glass and metal surfaces were cleaned in an ultrasonic bath with a solution of isopropyl alcohol and distilled water, followed by drying in a dust‐free laminar flow hood. Plastic and wooden surfaces were cleaned with a mild detergent, rinsed with distilled water, and air‐dried in a controlled environment. Agarose hydrogel substrates of varying concentrations (e.g., 0.08% and 0.15%) and incline (e.g., 60°) were meticulously cast to simulate a range of surface properties. Agarose hydrogels were prepared by dissolving agarose powder in distilled water, heating to 95 °C, and cooling to room temperature to achieve specific concentrations.^[^ ^82^ ^]^ The hydrogels were cast into molds under a laminar flow hood to ensure uniform thickness. Biological substrates were handled with precision to preserve their native characteristics. Sheep meat, fat, bone, and fish eyes were obtained from reliable fresh sources, adhering to University College London ethical regulations, and kept under controlled refrigeration conditions to preserve their integrity. They were trimmed to uniform dimensions and stored at 4 °C to preserve their integrity, while human skin and nail samples were sterilized using UV radiation. These detailed procedures, combined with consistent environmental controls, ensure that the substrate preparation process is both reproducible and scientifically robust.^[^ ^83^, ^84^ ^]^ Strict ethical considerations and sterilization procedures were scrupulously followed when collecting human skin, nail, and samples.

High‐speed camera experiments were conducted by directing droplets onto the meticulously prepared substrates and capturing the resulting interactions. The careful preparations ensured the reliability and consistency of the experimental data. This was essential for analyzing the complex dynamics of droplet impact and splashing across the range of substrates and experimental conditions considered.

### 2.3 Experimental Approach: Droplet Splashing

Droplet splashing dynamics was captured using the high‐speed cameras. Recorded images were analyzed using MATLAB (R2022b, The MathWorks Inc.) to ensure their comprehensive and accurate evaluation. Our bespoke MATLAB code analyzed the droplet impact and splashing within the image sequences. Splashed spot diameters and velocities were detected and calculated by overlaying recognized spots onto the thresholded images. Histograms were generated for the spot diameters and velocities. The software also reconstructed the trajectory paths of individual spots, including secondary droplets. Figure 2c illustrates an image analysis for a water droplet impacting onto a water layer and the velocity vectors of the splashed secondary droplets at times 7.5 and 25 ms, respectively, after impact, with We = 1200 and a droplet diameter of 3 mm. The choice of a 3 mm diameter was guided by its relevance to practical scenarios, such as medical diagnostics and environmental studies, where this droplet size is frequently encountered. Our findings highlight the sensitivity of the detection method to secondary droplet formation, particularly for fine and larger droplet sizes exceeding 100 μm. The algorithm used for analyzing droplet growth and movement was customized for analyzing image sequences and tracking the growth and movement of individual droplets as spots over time. The calculations involve parameters such as the pixel‐to‐millimeter scale and the customized frame rate. The algorithm then analyzed each image to track key parameters, like the spot diameter, area, and velocity. Morphological procedures were applied to improve the binary picture. Binarization was based on a predetermined threshold and images were converted to grayscale. Figure 2d illustrates the image analysis of a water droplet impacting onto a dry plastic substrate and then spreading until relaxation occurs. The droplet diameter and area increase over a time period ranging from 0.4 to 5.4 ms and subsequently decrease gradually until 113.5 ms.

Polygonal regions of interest (ROI) were analyzed in the image sequences to produce histograms of the red, green, and blue color channel intensities. The binary masks generated by the code based on these drawn regions effectively isolate the pixels contained within the ROIs. Histograms were then computed for each color channel within the ROIs, normalizing them to unit total count to represent probabilities. This approach proved to be useful when investigating droplet impact scenarios involving color‐based contamination, as shown in Figure 2e–i.

## 3 Results and Discussion

### 3.1 Effects of Contamination on Secondary Droplet Spectra

The images in Figure 2f,h depict the impact of a 3 mm diameter water droplet onto a water layer with We = 1700. The moment of contact corresponds to time zero. The images in Figure 2f follow the progression of a noncolored droplet impacting onto a colored water layer. The first frame displays the noncolored droplet; in the second frame at time 20.78 ms, the crown pattern becomes red. The zoomed‐in view in the third frame reveals the rim boundary and the formation of secondary droplets. This image sequence resembles the observation in Figure 2h, which shows red droplets formed after the impact of a red droplet onto a noncolored water layer; the image of the crown corresponds to 21.32 ms. The rim and secondary droplets are captured as in Figure 2f. In Figure 2g,i, the red‐channel probability is evidently significantly higher than for the other color channels. This observation implies that red dominates in the resulting secondary droplets. The increased probability of the red channel indicates that, when a red droplet impacts or when a red water layer is involved, it produces a distinctive red intensity in the secondary droplets. This finding suggests that the color of the initial droplet or the medium it impacts upon significantly determines the color characteristics of the splashed secondary droplets. The production of a mixed rim upon splashing is mainly due to the complex process of mixing between the impacting droplet and the underlying layer, which is the primary cause of the higher probability of the red channel in Figure 2g,i. Essentially, our experiments simulate contamination dynamics by monitoring the localization of the red color. This effectively mimicked the possibility of secondary droplet contamination when the impacting droplet or contact layer carries a specific color, which is then transferred to the splashed secondary droplets.

### 3.2 Droplet‐Impact Splashing Dynamics on Biological Fluids Layers

**Figure 3:**
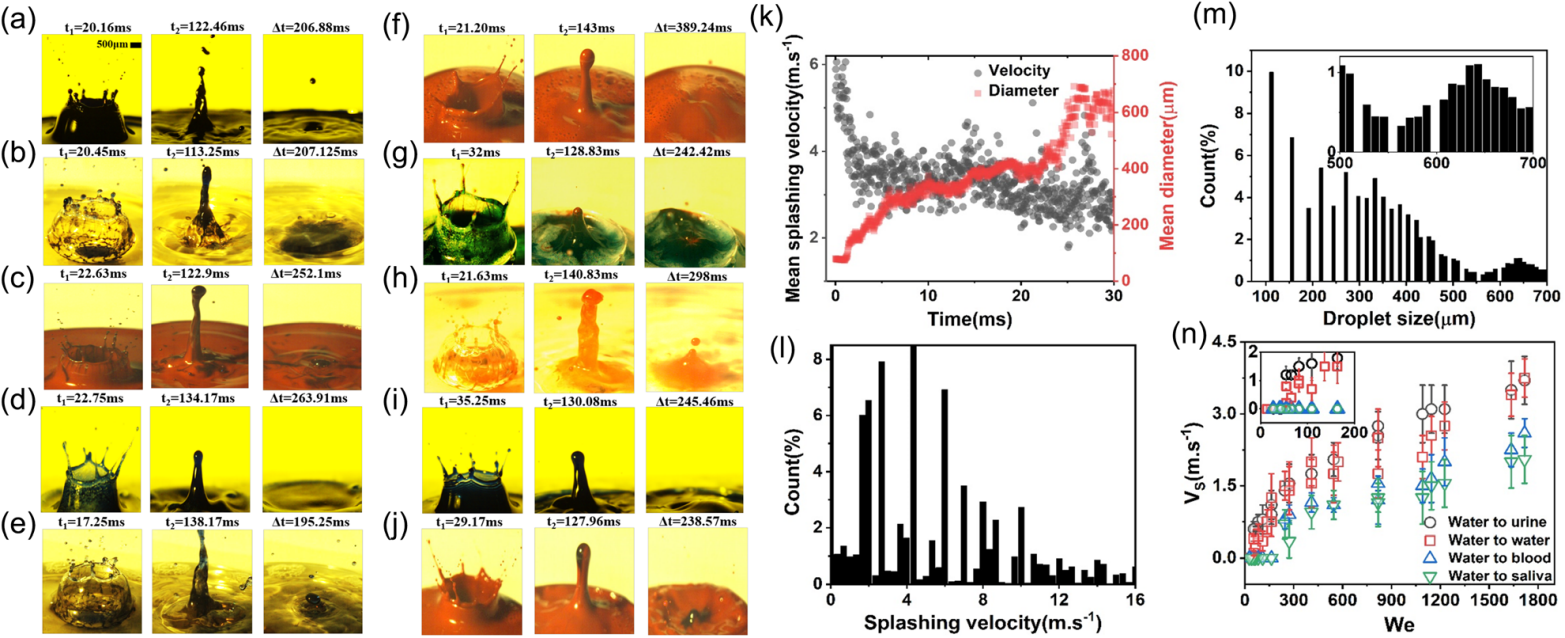
Droplet‐impact splashing on biological fluids, imaged over time. a–j) Splashes resulting from a 3.4 mm diameter droplet impacting various biological fluids at 6.4 m s^−1^: water droplet impacting onto a) water, b) urine, c) blood, and d) saliva surfaces; urine droplet impacting onto e) urine liquid; blood droplet impacting onto f) blood, g) saliva, and h) urine; and saliva impacting on i) saliva and j) blood fluids under identical experimental conditions. The qualitative characteristics of crown growth (*t*_1_), jetting (*t*_2_), and relaxation time (Δ(*t*)) are apparent. k) Plots of the mean splashing velocity and diameter of a secondary droplet over time for a water droplet impacting onto water. Histogram are shown for l) the splashing velocities and m) sizes of secondary droplets, following the impact of a water droplet onto water. n) Mean splashing velocity plotted as a function of We for a water droplet impacting onto water, urine, blood, or saliva.

**Figure** **3** analyzes the droplet‐impact splashing phenomenon when impacting onto various biological fluids. When a 3.4 mm diameter droplet impacts onto various fluid layers with We = 1700, the splashing features, such as growth and collapse (*t*_1_), jetting (*t*_2_), and relaxation time (Δ(*t*)), display unique behaviors. In the case of water droplets impacting onto various fluids (Figure 3a–d), the crown and rim generation time for splashing decreases as the fluid's viscosity increases. Water droplets impacting onto urine (Figure 3b) exhibit the shortest rim generation time (Δ(*t*) = 20.45 ms), while water impacting onto saliva (Figure 3d) yields the longest rim generation time (Δ(*t*) = 22.75 ms) owing to the greater viscosity of saliva.

A similar trend is observed for nonwater droplets impacting onto different fluids (Figure 3e–j). Figure 3f,g shows a longer rim generation time for blood droplets impacting onto blood (Δ(*t*) = 21.20 ms) and saliva (Δ(*t*) = 32 ms), while the shortest rim generation time (Δ(*t*) = 12.63 ms) occurs when blood droplets impact onto urine (Figure 3h). These variations result from the fluid viscosities: saliva and blood are more viscous than urine. These results highlight the significant influence of fluid viscosity on droplet‐impact splashing dynamics, with higher‐viscosity fluids producing shorter times for growth, collapsing, jetting, and relaxation. Understanding these dynamics is crucial for applications involving droplets in medical and biological contexts, where controlling splashing has implications for infection control and safety. Thus, viscosity significantly influences droplet impact dynamics and the subsequent sheet formation on biological fluid layers. In cases of relatively low viscosity, jets formed by the impact occur almost simultaneously and quickly merge, rapidly forming a single sheet. However, a greater viscosity delays and weakens the substrate jet. These findings are consistent with previous observations.^[^ ^64^, ^85^, ^86^ ^]^

The analysis presented in Figure 3k provides insights into the mean splashing velocity and the diameter of secondary droplets when a water droplet impacts onto water (We = 1700). On average, the splashing velocity of secondary droplets is ≈3.7 m s^−1^, with a mean diameter of 280 μm. In conjunction with this analysis, the histograms in Figure 3l,m show the distributions of the splashing velocities and the secondary droplet sizes, respectively, for a water droplet impacting onto water. The histograms reflect the diverse splashing characteristics that are particularly valuable in contexts where the generation of aerosols and airborne particles is a concern. The data reveal that 30% of the splashing events occur within a velocity range of 2–6 m s^−1^, and 40% of secondary droplets splash are within the size range of 100–300 μm. These results clarify the dynamics of droplet impact, particularly for the cases when We = 1700. Importantly, these data suggest that droplets of size 100–300 μm and velocities of 2–6 m s^−1^ can interact with other surfaces or fluid layers, potentially producing thin droplets on the scale of airborne particles and aerosol droplets. The potential for generating aerosols (*<*20 μm), fine droplets (20–200 μm), or larger droplets (*>*200 μm) depends on the magnitude of the forces acting on the water films, as shown in previous studies.^[^ ^9^, ^10^ ^]^

The correlation between the mean splashing velocity and We for droplets impacting onto water, urine, blood, and saliva layers is displayed in Figure 3n. The experiments involved droplets of diameter 3 mm released from different heights (ranging from 0.05 to 2.1 m) above the target surfaces. This observation emphasizes the sensitivity of the detection method to secondary droplets in the fine and larger droplet size ranges, typically greater than ≈100 μm. Key observations emerge from the analysis of the captured images. First, no droplets were detected for blood and saliva layers for We* < *300. The absence of splashing within this range results from the higher viscosities and densities. Second, a similar trend was observed for the water and urine layers, where no droplets were present for We* < *70. The mean velocity of the splashed droplets increased nonlinearly with increasing We. Urine and water displayed a greater rate of increase compared to blood and saliva. Notably, the maximum mean velocity of the secondary droplets was 2.5 m s^−1^ for the blood and saliva layers, and reached 4 m s^−1^ for the water and urine layers. These results highlight the fact that fluid properties, particularly the viscosity, dictate the droplet splashing dynamics, with potential implications for infection control and safety measures in medical and biological contexts. The analysis of Figure 3 underscores the significance of non‐Newtonian fluid properties (e.g., in the case of saliva and blood). Such non‐Newtonian fluids exhibit unique rheological behavior characterized by viscosity changes in response to the shear rate or stress.^[^ ^47^, ^78^, ^80^ ^]^ Blood viscosity tends to increase with shear rate, a phenomenon known as shear‐thickening.^[^ ^47^, ^80^ ^]^ This property influences the splashing dynamics, as observed in the analysis, where blood exhibits relatively shorter crown growth, collapse, and jetting times when impacting onto other fluids. Conversely, saliva displays shear‐thinning behavior, featuring extended times for these same qualitative characteristics.^[^ ^78^ ^]^ The influence of fluid viscosity, controlled by their non‐Newtonian properties, is evident in the varying splashing velocities and secondary droplet sizes observed across different biological fluids. These findings emphasize the importance of the rheological properties of non‐Newtonian biological fluids when analyzing droplet impact and splashing behavior, particularly in biomedical and clinical applications where infection control and safety are critical.

### 3.3 Dynamics of Droplet Impact onto Various Substrates in Medical Environments

A wide selection of materials was chosen for their specific hydrophilic, hydrophobic, and roughness properties, serving various functions in medical and cleanroom settings. These materials included metals (often used in surgical instruments for their durability and ease of sterilization) and glass (commonly found in transparent containers and laboratory equipment). Tissue and paper materials are also frequently employed to fabricate disposable medical supplies, while wood is used in furniture or structural elements within laboratory and clinical spaces. The capacity of hydrogels and foams for absorbing water and biological fluids make them choice materials for various applications (e.g., cleaning, wound dressings, and drug delivery systems).^[^ ^87^ ^]^ Fabrics and textiles provide comfort and breathability to hospital linens and patients. Plastic materials are highly versatile and are the main material in medical items like syringes, tubing, and implantable components.^[^ ^88^ ^]^ An understanding of how droplets interact with these diverse materials, each characterized by unique properties, provides insights for maintaining hygiene, preventing contamination, enhancing patient care, and ensuring the safety and efficacy of medical procedures in medical and cleanroom spaces.^[^ ^89^ ^]^

**Figure 4:**
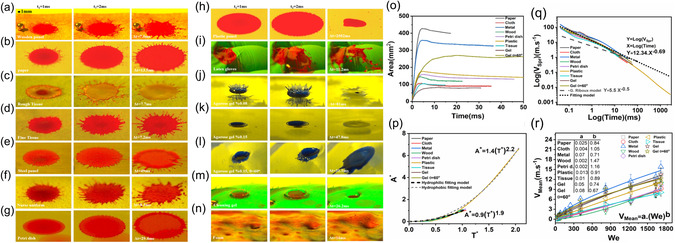
Droplet impact behavior on diverse medical substrates. a) Wooden panel, b) paper, c) rough tissue, d) fine tissue, e) steel panel, f) nurse uniform, g) petri dish, h) plastic panel, i) Latex gloves, j) agarose gel with 0.08% Concentration, k) agarose gel with 0.15% concentration, l) agarose gel with 0.15% concentration (*θ *= 60°), m) cleaning gel, n) foam. Each substrate, labeled from a) to n), is captured at three different time points after droplet contact with the substrate (*t*_0_ = 0): *t*_1_ = 1 ms, *t*_2_ = 2 ms, and Δ(*t*) during relaxation when the fluid stabilizes with We = 1700. o) Plots of the change in fluid area over time when a droplet impacts onto various substrates. The data captures the evolution of the fluid area until it reaches a relaxed state where no further movement occurs. p) Plots of dimensionless area and time. The dimensionless area is defined as the area divided by the final area, while dimensionless time is time divided by the final time. q) Plots of logarithmic spreading velocity of a fluid on different substrates. r) Plot of the mean velocity of fluid spreading on a substrate as a function of We.

**Figure** **4** investigates the dynamics of droplet impact onto a range of surfaces commonly encountered in medical and cleanroom environments. These observations highlight the essential role of the material properties in shaping the interactions involved. The substrates considered include wood, paper, tissue, metal, plastic, gel, and latex. The surface roughness and hydrophobic or hydrophilic properties define characteristics that are unique to each substrate. The resulting diversity underscores the multifaceted nature of droplet–surface interactions under different We conditions. Hydrophilic surfaces, such as tissue, paper, and wood, favor rapid stabilization and limited spreading thanks to their efficient water‐absorption capabilities.^[^ ^90^, ^91^ ^]^ In contrast, hydrophobic surfaces like petri dishes, metal, and plastic panels (Figure 3e–h) favor delayed relaxation and more extensive spreading areas.^[^ ^92^, ^93^, ^94^ ^]^

The contact angle of the impacting droplet is the key determinant of these distinct behaviors. Hydrophilic surfaces are associated with a smaller contact angle, allowing for rapid wetting and absorption. In contrast, hydrophobic surfaces induce larger contact angles, resulting in delayed wetting and extended relaxation times. This contact angle effect, in conjunction with the surface roughness, significantly influences the droplet splashing trend. This is demonstrated in Figure 4a–f, where increasing the roughness intensifies splashing events and their spreading area (Figure 4o). Significantly, materials with diverse roughness profiles exhibit distinctive splashing potentials, with paper and metal consistently manifesting the highest spreading velocities under varying We conditions (Figure 4r). This is underscored by the common scaling behavior, depicted in Figure 4p. The curves representing different substrates converge, indicating a common scaling framework governing droplet impact and spreading. Surface roughness provides insights into the detailed interplay between material properties and droplet dynamics across various surfaces in medical and cleanroom settings. Figure 4i reveals a splashing mechanism inherent to latex material, primarily attributed to the surface inclination and the concave and convex features formed in latex gloves. This unique phenomenon produces a distinctive splashing pattern that deviates significantly from that typically observed on flat or uniformly textured surfaces. The inclination and irregularities on the latex glove surface play an important role in determining the observed splashing behavior, underlining the importance of the surface geometry in dictating splashing dynamics and the associated contamination risks. Figure 4j,k displays the remarkable characteristics of agarose hydrogel with concentrations of 0.08% and 0.15%, respectively. The behavior of the impacting droplets is intrinsically linked to the material's viscosity, a direct consequence of the agarose hydrogels’ viscoelastic properties. The less viscous 0.08% hydrogel allows sheet and rim formation without fragmentation at the rim boundary, effectively suppressing the production of secondary droplets. Conversely, the denser 0.15% hydrogel structure inhibits secondary droplet formation. Furthermore, introducing a 60° slope (Figure 4l) accentuates the production of secondary droplets because of the altered flow dynamics. Figure 4i,l highlights the fundamental importance of the surface inclination in governing droplet dynamics. The inclined surface extends the droplet relaxation time, facilitating a faster and more extensive flow across the latex gloves and hydrogel surfaces. A comparative analysis between Figure 4k,l clearly reveals an increase in splashing and secondary droplet formation for the inclined hydrogel. This intricate interplay between surface inclination and the hydrogel viscoelastic properties significantly contributes to the generation of secondary droplets and to the observed splashing behavior. Notably, these effects are particularly prominent on solid surfaces like those of latex gloves and an inclined hydrogel. In summary, these results underscore the complex interplay between the surface geometry, material properties, and droplet dynamics. The surface inclination tends to enhance splashing and the creation of secondary droplets, whereas the viscoelastic characteristics of the hydrogel mitigate the splashing behavior. The overall outcome is a delicate balance that significantly influences overall droplet dynamics. These insights have substantial implications for applications in medical and cleanroom environments, where the precise control of droplet interactions is essential for maintaining hygiene and safety standards. The mean spreading velocity is plotted as a function of We in Figure 4r. The corresponding experiments involved 3 mm diameter water droplets falling from varying heights (ranging from 0.05 to 2.1 m), yielding a broad range of impact velocities. The material's surface roughness profoundly influences the droplet behavior, as demonstrated in Figure 4a–f. On rough surfaces like wood, paper, tissue, and metal, a droplet impacting with We = 1700 causes the splashing of secondary droplets at a mean spreading velocity of up to 18 mm s^−1^. Conversely, there is no splashing for We values below 200, in which case the droplets instead predominantly spread over the surface with a mean spreading velocity below 3 mm s^−1^. The relationship between We and the impact velocity is nonlinear across all the tested materials. These behaviors highlight the sensitivity of splashing to the surface roughness and indicate that even minor variations significantly affect droplet dynamics. The effect of the surface roughness is further emphasized by examining tissue with varying degrees of roughness in Figure 4c,d. As the surface roughness increases, the propensity for splashing grows, clearly demonstrating the significant influence of the surface texture on droplet impact dynamics.^[^ ^60^, ^61^, ^95^ ^]^ The increase in the mean velocity for each We is relatively consistent across different surfaces, spanning from the lowest mean velocity for tissue to the higher mean velocity observed for the metal surface.

The results in Figure 4 suggest that surface properties significantly influence droplet impact dynamics across various We conditions. Collectively, these insights provide useful guidance for optimizing material selection and for comprehending splashing dynamics across diverse practical scenarios in medical and healthcare settings. Additionally, the findings emphasize the crucial interplay between surface characteristics and droplet dynamics; these observations are of direct practical relevance.

This study analyzed the splashing behavior of droplets impacting onto various surfaces using both experimental data and established theoretical models. Specifically, we employed the formula *V*_t_ = 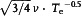, proposed by Riboux et al.^[^ ^70^ ^]^ to predict the critical impact velocity for droplet splashing. In this equation, *v* represents the impact velocity and *T*_e_ is the ejection time. Using this model, we calculated the spreading velocity of droplets over time and derived a logarithmic relationship. The experimental data in Figure 4q were fit to the curve *Y *= 12.34 *X* ^0.69^, which closely approximates Riboux's logarithmic model, *Y *= 5.5 *X* ^0.5^. Despite the differences in fluid properties and We values, our experiments involved droplets with We in the range of 10–1000. The two curves are in reasonable agreement. The variation in the constants can be attributed to differences in the liquid properties and the distinct order of magnitude of We. Additionally, this logarithmic relationship serves as a predictive tool for estimating the spreading velocity over time, providing valuable insights into the droplet dynamics for different fluids and surfaces.

We also examined the spreading behavior of droplets on hydrophilic and hydrophobic substrates by analyzing the nondimensional spreading area *A* ^*^ as a function of the nondimensional time *T* ^*^. In Figure 4p, we present two distinct models. For hydrophilic substrates like paper, wood, and cloth, the spreading area obeys *A* ^*^ = 0.9 (*T* ^*^)^1.9^, while for hydrophobic surfaces such as metal, petri dishes, and plastic, we have *A* ^*^ = 1.4 (*T* ^*^)^2.2^. These curves demonstrate the significant effect of surface wettability on droplet spreading behavior. For example, on hydrophilic surfaces, the spreading area increases more slowly compared to hydrophobic surfaces, which exhibit a steeper power‐law behavior. Additionally, our detailed analysis found the mean spreading velocity to increase with We, with significant variations observed across different substrates. These findings are critical for understanding the influence of surface roughness and material properties on droplet impact and spreading behavior. They make robust quantitative predictions that are applicable to various biological and clinical contexts. Figure 4r analyzes the mean spreading velocity as a function of We, revealing that splashing behavior depends strongly on the substrate roughness. For instance, for We = 1700, rough surfaces like wood and paper induce secondary droplet splashing at mean spreading velocities of up to 18 mm s^−1^, whereas smoother surfaces exhibit no splashing below We = 200. Whereas substrate variability makes the extraction of a universal function relating the mean spreading velocity and We impractical, power‐fitted curves (*V*_Mean_ = *a* We^b^) provide substrate‐specific predictions. These quantitative analyses, which integrate surface roughness effects and the non‐Newtonian nature of droplet impact, provide a deeper understanding of droplet dynamics and broaden the applicability of our findings to practical scenarios.

### 3.4 Surface Effects on Droplet Impact onto Biological Substrates

**Figure 5:**
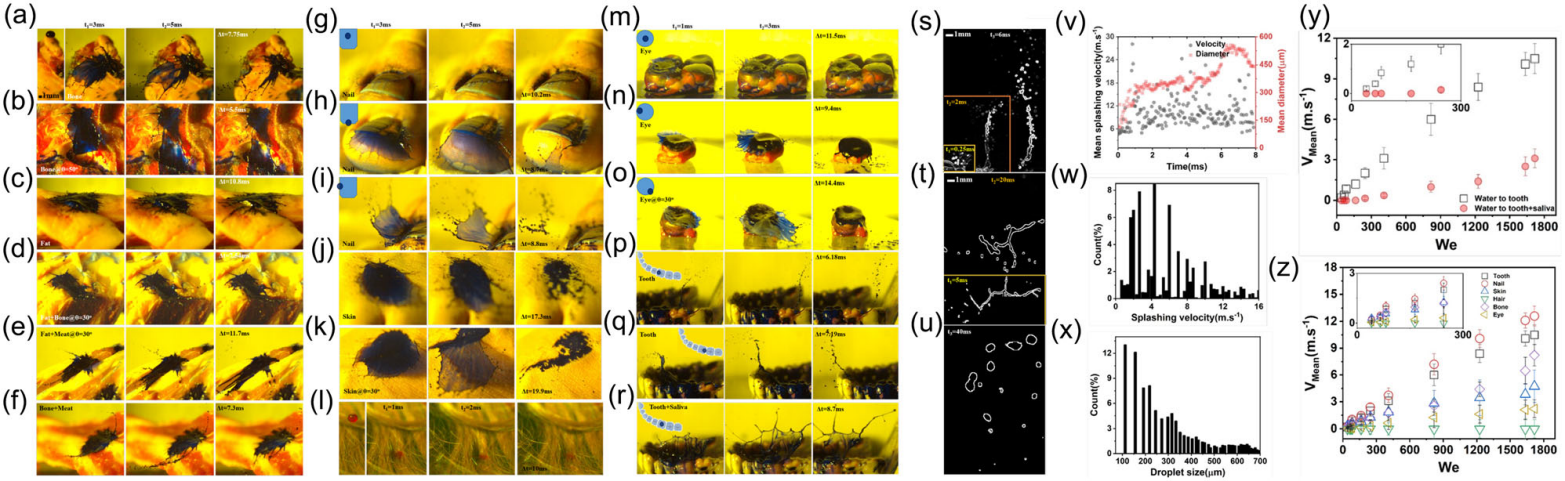
Water droplet behavior and splashing mechanisms observed on diverse biological substrates under varying conditions. Experiments were conducted with We = 1700, a droplet diameter of approximately *D *= 3 mm, and substrates including a) sheep bone, b) sheep bone inclined at *θ *= 50°, c) sheep fat, d) a substrate combining sheep bone and fat with *θ *= 30°, e) a substrate combining sheep meat and fat with *θ *= 30°, f) a substrate combining sheep bone and meat with *θ *= 30°, g) the center of a human nail, h) the front edge of a human nail, i) the region between human skin and a nail, j) human skin, and k) human skin with *θ *= 30°, l) a woman's hair, m) the center of a sheep's eye, n) the corner of a sheep's eye, o) the center of a sheep's eye with *θ *= 30°, p) the front and q) back parts of a dry molar tooth, and r) the central part of a molar tooth covered by a human saliva layer. Images for each substrate were captured at three time points after the droplets made contact with the substrate: *t*_0_ = 0, a–l) *t*_1_ = 3 ms and *t*_2_ = 5 ms; m–r) *t*_1_ = 1 ms and *t*_2_ = 3 ms, as well as Δ(*t*), the relaxation state when the fluid is stable and not flowing. s) Threshold images for the analysis of splashing water droplets at three time points: 0.25, 2, and 6 ms, based on the tests depicted in (p). t,u) Display threshold images used for the analysis of splashing droplets at time points 5, 20, and 40 ms, derived from tests conducted in images r). v) Mean splashing velocity and mean diameter, plotted versus time, utilizing data from the tests corresponding to images p). Histograms are shown for the relative distributions of w) the splashing velocity and x) the size of the splashed droplets. y) Mean velocity of secondary droplets plotted as a function of We, considering the impact on a dry tooth and on a tooth covered by a saliva layer. z) Mean velocity of secondary droplets as a function of We for water droplets impacting onto the different biological substrates featured in (a,i,k,l,m,p).

Secondary droplets produced by splashing during medical procedures may carry biological contaminants, including bacteria and viruses, potentially leading to disease transmission via air and surface contamination.^[^ ^28^, ^29^, ^96^ ^]^ Such splashing can originate from a range of substrates, including biological materials like those of bone, meat, fat, skin, hair, nails, eyes, and even teeth. Various experiments have been conducted to better understand and potentially mitigate this phenomenon. High‐speed cameras were employed to capture splashing behavior. For example, **Figure** **5**a–f depicts droplet impacts on sheep bone, meat, and fat surfaces for a range of incline angles.

These hydrophobic surfaces, particularly those of bone, exhibit distinctive spreading and splashing behaviors. Splashing and secondary droplet formation increase with incline angles and surface irregularities, especially those associated with bone. The phenomenon of splashing was observed on nail surfaces where the droplet impacted the concave region between the nail and the skin. Similar behavior is also apparent on the nail surfaces in Figure 5a–j. The edges of solid or soft substrates, such as nails and eyes, contribute to producing secondary droplets, as evidenced in Figure 5h,n. In contrast, the porous structure of hair prevents splashing (Figure 5l) by dissipating the energy of the impacting droplet. Different types of hair, with varying numbers of strands and varying roughness, yield different responses to droplet impact. Here, the experiment focused on droplet impact onto dry and smooth hair. Figure 5m–o displays droplet impacts on the soft surface of a fish eye cornea. Figure 5o suggests that the incline angle causes splashing. Droplets at the eye center produced secondary droplets, primarily because of the convex shape of the droplets on the cornea surface. Droplet impact tests were also performed on real tooth geometries. These tests considered real tooth surfaces, which interacted with uneven and rough surfaces, including the concave section of the crown—a potential saliva reservoir. The oral cavity's influence on splashes and aerosol flows was also considered. The experiments examined different parts of tooth 36 (ISO 3950):^[^ ^97^ ^]^ the front (Figure 5p), back (Figure 5q), and central parts covered by human saliva (Figure 5r). Figure 5p,q shows distinct splashing directions to be influenced by the concave structure of the crown and the oral cavity. Saliva, in addition to its role in preventing, combatting and protecting from disease, can collect in these cavities. As shown in Figure 5r, the introduction of natural human saliva has a discernible impact on the splashing behavior, primarily owing to its viscosity. Furthermore, the influence of the oral cavity on splashes and aerosol flows was considered when examining a real tooth. As a sample of image processing analysis, threshold images were generated to facilitate the analysis of splashing droplets (Figure 5s–x). Figure 5s displays threshold images used for analyzing splashing droplets at three time points 0.25, 2, and 6 ms, based on the tests featured in Figure 5p. Similarly, Figure 5t–n displays threshold images used to analyze splashing droplets at time points 5, 20, and 40 ms, for the tests represented in Figure 5r.

Figure 5v–z presents various aspects of the experimental results analysis. Figure 5v includes a plot of the mean splashing velocity and the mean diameter over time, derived from the tests featured in Figure 5p. Figure 5w,x shows normalized histograms of the splashing velocity and size of the splashed droplets. Figure 5y plots the mean velocity of secondary droplets as a function of We, considering the impact on a dry tooth and a tooth covered by a saliva layer. The presence of saliva on the tooth reduces the splashing velocity from 11 to 3 m s^−1^ for We = 1700. Finally, Figure 5z plots the mean velocity of secondary droplets versus We for water droplet impacts onto the different biological substrates depicted in Figure 5a–p. The experiments involved 3 mm diameter water droplets falling from various heights ranging from 0.05 to 2.1 m. Nail and tooth substrates exhibit higher splashing mean velocities, while eye surfaces yield lower velocities and hair produces no splashing droplets. This plot reveals how increasing the droplet impact velocity increases the splashing velocity of the secondary droplets. It also illustrates how variations in the surface geometry can influence and intensify splashing. Notably, no studies reported to date have investigated droplet impact onto biological substrates similar to the materials considered in this study (meat, bone, skin, eye, hair, etc.). The present work therefore constitutes a pioneering investigation in this field, providing valuable insights into splashing phenomena onto various biological substrates.

Figure 5 highlights the importance of the surface characteristics, e.g., the hydrophobicity, roughness, wetness, and contact angle of droplets on a surface, for controlling splashing behavior. Notably, the presence of unevenness arising from surface convexities and concavities, often attributed to the nonuniformity and inhomogeneity of materials like bone, plays an important role in initiating and amplifying the splashing process. This insight into the interplay between surface properties and droplet dynamics contributes to our ability to control and mitigate splashing events. This is particularly important in high‐risk environments such as medical settings, where minimizing disease transmission is a priority. Furthermore, these findings pave the way for the development of advanced materials and design strategies to enhance the safety and effectiveness of various applications where droplet management is critical.

### 3.5 Effect of the Surface Roughness on the Splashed Secondary Droplets

**Figure 6:**
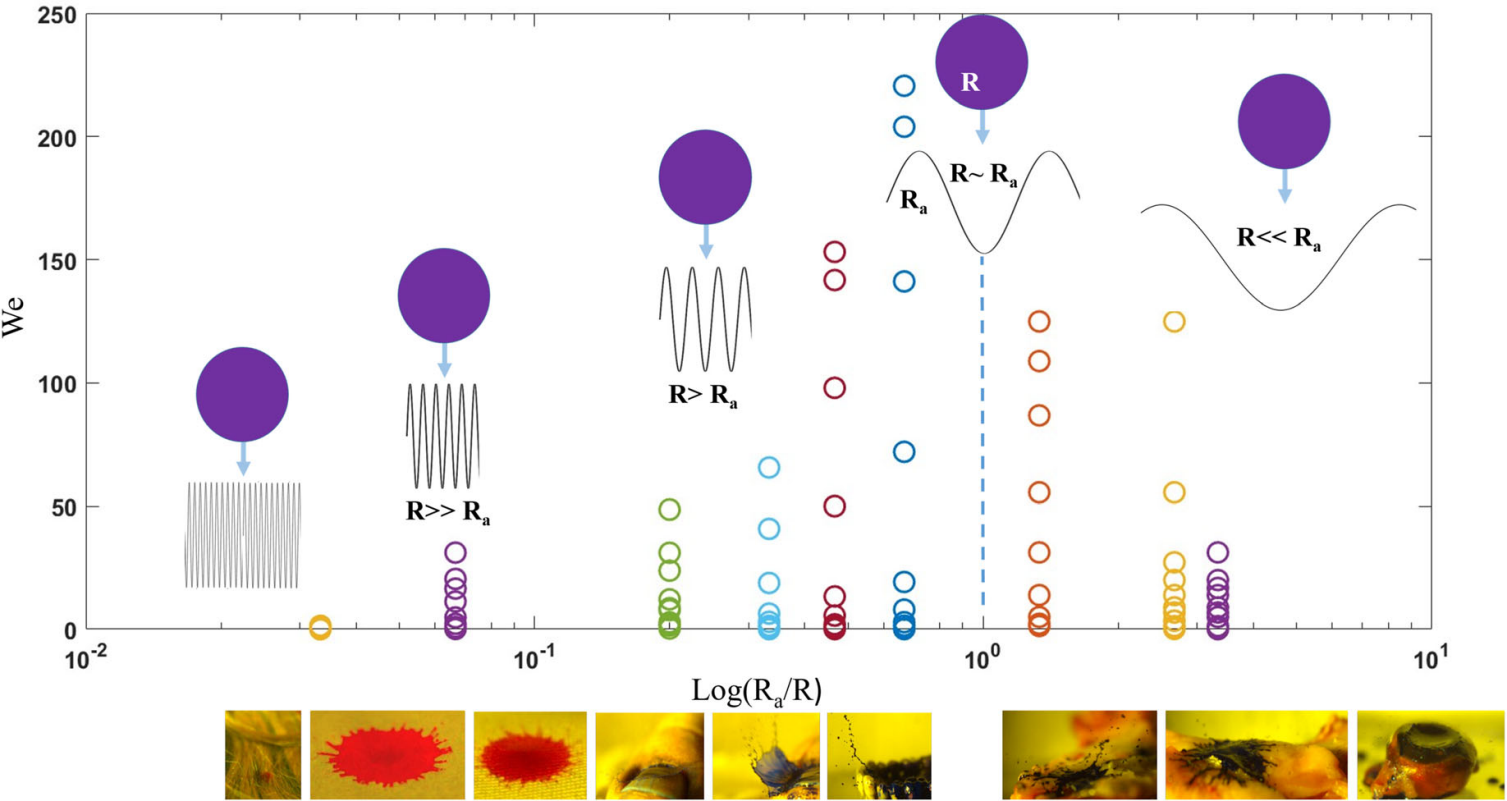
Effect of the surface roughness scale on the splashed secondary droplet. We is plotted as a function of the logarithmic normalized roughness, *R*_a_ */R*, where *R*_a_ quantifies the roughness and *R* is the droplet radius.

The experiments depicted in Figure 3, 4, 5 provide insights into the dynamics of droplet impact onto various substrates, including those commonly found in medical and clinical environments. This section examines the source of splashing described above, which leads to the formation of secondary and fine droplets. As illustrated in Figure 4, the primary driver of droplet spreading and splashing upon impact is the substrate roughness. The roughness induces variations in droplet size and kinetic energy, and hence in the droplet velocities both in air and on the surface. **Figure** **6** presents an analysis in terms of We. It is based on the normalized roughness that characterizes secondary droplets’ behavior with respect to the mean velocity and the diameter, as observed in Figure 4, 5. We is defined as *ρD*′*V*′^2^ */σ*, where *D*′ represents the mean secondary droplet diameter and *V*′ is the mean velocity of secondary droplets. The normalized roughness is defined as *R*_a_ divided by the droplet radius *R*.

The roughness of biological material surfaces, such as those of human nail, skin, and bone, varies across different studies and measurement techniques. For human nail, *R*_a_ ranges from 0.1 to 0.2 μm, reflecting microscopic surface irregularities.^[^ ^98^ ^]^ Human skin, on the other hand, displays varying roughness, with *R*_a_ typically ranging from 10 to 100 μm, contingent on factors such as location and skin health.^[^ ^99^, ^100^ ^]^ Human bone surfaces, specifically those of trabecular bone, are notably rougher, exhibiting roughness values *R*_a_ = 4–5 μm.^[^ ^101^ ^]^ Additionally, hair strands collectively have roughness values ranging from ≈0.05 to 0.5 μm,^[^ ^102^, ^103^ ^]^ influenced by factors such as hair type, care routines, environmental exposure, and potential hair damage or treatments. Moreover, the roughness of various nonbiological substrates also plays a crucial role in controlling droplet dynamics. Wooden surfaces have *R*_a_ values ranging from 0.5 to 20 μm depending on the wood species and surface preparation.^[^ ^104^, ^105^ ^]^ Metal surfaces, depending on factors like the alloy composition and the machining process, exhibit *R*_a_ values ranging from 0.005 to 2 μm.^[^ ^106^ ^]^ Paper surfaces have typical *R*_a_ values between 0.05 and 3 μm, influenced by the paper type and manufacturing methods.^[^ ^107^ ^]^ Importantly, tooth surfaces also display variable roughness, influenced by factors such as the tooth type, the location within the oral cavity, oral hygiene practices, dietary habits, and dental treatments. On average, *R*_a_ for human tooth surfaces ranges from a few micrometers to around 10 μm. The enamel on the chewing surfaces of molars tends to be smoother than the enamel on the sides of teeth, contributing to variations in roughness. Dental practices, including tooth brushing, cleaning, and treatments like polishing and restorations, can also influence the tooth surface roughness.^[^ ^108^, ^109^ ^]^

Figure 6 shows further details of the influence of surface roughness on splashing on the micro‐ and macroscales. When *R*_a_ *≪ R*, (corresponding to microscopic roughness as displayed by, e.g., hair, glass, paper, fabric, metal, bone, nail, skin, and wood), droplets break up on impact and produce secondary droplets. The impact velocities of the secondary droplets increase with *R*_a_. As *R*_a_ approaches *R*, the surface's geometric curvature dominates the droplet dynamics. Surfaces like those of skin, nails, and teeth, which contain concavities and convexities, direct the droplet's flow based on their geometry. This flow may transition from a splash to no contact with the surface (surface‐independent flow^[^ ^110^ ^]^); finally, the droplet breaks up into secondary droplets. Notably, the unevenness of the surfaces of skin, tooth, the skin‐nail regions, and sheep meat substrates fall into this category, typically with 0.35 *< R*_a_ */R < *1. In cases where *R*_a_ ≫ *R*, (i.e., surfaces with a significant curvature compared to microscopic roughness), the surface's curvature becomes the primary driver of droplet movement and splashing behavior. As shown in Figure 6, droplet impact on different combinations of sheep bone, meat, and fat surfaces (Figure 5) falls within this category. These surfaces exhibit reduced splashing velocities because of the increased curvature in their concave geometries. The effects of geometric surface curvature, arising from macroscopic roughness, and of microscopic roughness are crucial and noteworthy results in this study. This finding is significant in the context of medical and nonmedical applications, particularly those that require the control of droplet splashing effects on surfaces under diverse conditions. The development of advanced biomaterials and their design techniques can also improve strategies for minimizing droplet splashing and for improving safety in healthcare. Moreover, these insights can be applied to the production of fine aerosol particles, a highly relevant process in pharmaceutical applications, spray coating, and the development of advanced drug delivery systems.

### 3.6 Using Advanced Biomaterial Characterizations to Control Droplet Splashing in Medical Settings

Our results show that surface materials with minimal macro‐ and microscopic roughness can minimize splashing and reduce the formation of secondary droplets in medical and clinical spaces. Especially in medical environments, advanced biomaterial coatings provide an innovative approach to decreasing the chance of splashing and the spread of contaminants on surfaces. Flexible materials that are compatible and customizable for specific clinical environments can be created using biomaterial characterization. By combining experimental results of droplet splashing with appropriate features related to medical applications, this multidisciplinary method improves the development of biomaterials to provide acceptable solutions for reducing droplet splashing. The compatibility of advanced biomaterials with human organs is essential for their effectiveness at minimizing droplet splashing during medical interventions.^[^ ^111^ ^]^ A biomaterial's characteristics are essential to ensure its effective compatibility with a physiological environment. Biocompatibility is essential for preventing adverse responses, and conformability and flexibility are necessary to adapt to the different physiological organ forms and to reduce the possibility of splashing on curved surfaces.^[^ ^112^, ^113^ ^]^ In addition to preserving organ health, controlling membrane porosity and permeability helps to avoid splashing. Durability guarantees continued protection during treatments, while sterility is essential for preventing infections during direct organ contact.^[^ ^114^ ^]^ Particularly in dynamic situations, adhesive characteristics favor biomaterial adhesion, while degradability enables safe breakdown after treatment.^[^ ^112^ ^]^ Furthermore, stability and damping properties, which determine a biomaterial's capacity to absorb and release impact energy, are governed by its viscoelastic characteristics.^[^ ^115^ ^]^ Radiolucency, or transparency, is still necessary for visibility during imaging procedures.^[^ ^116^ ^]^

In medical settings, droplet splashing must be minimized by considering the biomaterial subsurface properties in addition to their surface characteristics. Our results show that the increased viscosity of a biofilm can be important. This is particularly true for droplets impacting onto blood and saliva biolayers. This highlights the role of increased viscosity in reducing the likelihood of splashing. To reduce the probability of splashing during medical operations, advanced biomaterials must improve subsurface characteristics and display adjustable rheological properties in addition to surface roughness and curvature.^[^ ^117^, ^118^ ^]^ This approach guarantees a more efficient decrease of contaminant spreading, especially in situations involving biofilms and other biological materials.

The physical form of a biomaterial (e.g., liquid or powder) presents a further significant challenge. The required form depends on the specific needs of the surgery. For instance, a liquid biomaterial may achieve greater adhesion and coverage on oral surfaces during dental operations, but a powder‐based formulation may be more appropriate for body plastic surgery techniques.^[^ ^119^ ^]^ In addition, biomaterials should be temperature‐adaptable for compatibility with the body temperature during medical operations. The ease of preparation, application, and cleaning of biomaterials, both during and after a surgical procedure, is an essential benefit. Quick and simple biomaterial preparation protocols guarantee their timely application and facilitate therapy.^[^ ^120^ ^]^

Taken together, these characteristics make cutting‐edge biomaterials more successful at reducing splashing and promoting organ compatibility, thereby improving their safety and performance in medical settings.

## 4 Conclusion

Building upon previous studies,^[^ ^2^, ^61^, ^68^, ^86^ ^]^ this work highlights the intricate dynamics of droplet splashing after impact onto a wide array of biological and medical materials that are relevant to clinical settings. The results on droplet spectra underscore the significance of controlling this phenomenon with regard to the probability of contaminant transmission through splashing originating from both droplet and biofilm sources. Our key findings highlight the significant role of non‐Newtonian fluid properties, emphasizing the effect of viscosity on splashing dynamics in the context of biological fluids. Surface properties, including roughness and hydrophobicity, emerge as critical factors influencing droplet impact, thus providing valuable guidance for material selection in healthcare environments. A notable feature of our work is the thorough examination of the effects of *R*_a_ */R* on splashing. This highlights the significance of geometric curvature and microscopic roughness for droplet dynamics, consistent with other findings relating to droplet impact on concave and curved substrates.^[^ ^68^, ^69^ ^]^ This distinct viewpoint deepens our knowledge of surface dynamics in biomaterials. The particular contribution of this study, in relation to previous works,^[^ ^42^, ^52^, ^61^, ^62^, ^68^, ^121^ ^]^ is its combined consideration of rheology and surface features in the context of splashing on biological and biofilm substrates. This highlights the relevance of biomaterials for healthcare applications and provides guidance for developing efficient droplet‐management solutions. Furthermore, the effect of a dominant surface roughness, demonstrated experimentally, highlights the need for tailored surface materials in clinical environments. Future research should focus specifically on the particular implications of these results for biomaterial applications, especially in relation to biofilms and other biological materials, for minimizing the risk of contaminant transmission.^[^ ^9^, ^18^, ^19^, ^32^ ^]^ Improving safety measures in medical settings will require an extensive study of the interaction between fluid dynamics and surface characteristics in more intricate biological situations. Furthermore, investigating cutting‐edge biomaterials and design techniques can ultimately improve methods for limiting droplet splashing and promote safer medical procedures.

## Conflict of Interest

The authors declare no conflict of interest.

## Data Availability

The data that support the findings of this study are available from the corresponding author upon reasonable request.
